# AI Algorithm to Predict Acute Coronary Syndrome in Prehospital Cardiac Care: Retrospective Cohort Study

**DOI:** 10.2196/51375

**Published:** 2023-10-31

**Authors:** Enrico de Koning, Yvette van der Haas, Saguna Saguna, Esmee Stoop, Jan Bosch, Saskia Beeres, Martin Schalij, Mark Boogers

**Affiliations:** 1 Cardiology Department Leiden University Medical Center Leiden Netherlands; 2 Lulea University of Technology Lulea Sweden; 3 Clinical AI and Research lab Leiden University Medical Center Leiden Netherlands; 4 Research and Development, Regional Ambulance Service Hollands-Midden Leiden Netherlands

**Keywords:** cardiology, acute coronary syndrome, Hollands Midden Acute Regional Triage–cardiology, prehospital, triage, artificial intelligence, natural language processing, angina, algorithm, overcrowding, emergency department, clinical decision-making, emergency medical service, paramedics

## Abstract

**Background:**

Overcrowding of hospitals and emergency departments (EDs) is a growing problem. However, not all ED consultations are necessary. For example, 80% of patients in the ED with chest pain do not have an acute coronary syndrome (ACS). Artificial intelligence (AI) is useful in analyzing (medical) data, and might aid health care workers in prehospital clinical decision-making before patients are presented to the hospital.

**Objective:**

The aim of this study was to develop an AI model which would be able to predict ACS before patients visit the ED. The model retrospectively analyzed prehospital data acquired by emergency medical services' nurse paramedics.

**Methods:**

Patients presenting to the emergency medical services with symptoms suggestive of ACS between September 2018 and September 2020 were included. An AI model using a supervised text classification algorithm was developed to analyze data. Data were analyzed for all 7458 patients (mean 68, SD 15 years, 54% men). Specificity, sensitivity, positive predictive value (PPV), and negative predictive value (NPV) were calculated for control and intervention groups. At first, a machine learning (ML) algorithm (or model) was chosen; afterward, the features needed were selected and then the model was tested and improved using iterative evaluation and in a further step through hyperparameter tuning. Finally, a method was selected to explain the final AI model.

**Results:**

The AI model had a specificity of 11% and a sensitivity of 99.5% whereas usual care had a specificity of 1% and a sensitivity of 99.5%. The PPV of the AI model was 15% and the NPV was 99%. The PPV of usual care was 13% and the NPV was 94%.

**Conclusions:**

The AI model was able to predict ACS based on retrospective data from the prehospital setting. It led to an increase in specificity (from 1% to 11%) and NPV (from 94% to 99%) when compared to usual care, with a similar sensitivity. Due to the retrospective nature of this study and the singular focus on ACS it should be seen as a proof-of-concept. Other (possibly life-threatening) diagnoses were not analyzed. Future prospective validation is necessary before implementation.

## Introduction

Overcrowding of emergency departments (ED) and hospitals is a concerning problem in many countries and is associated with increased mortality, delays in the initiation of critical care and dissatisfied patients and health care workers [1,2]. The causes of overcrowding are multifactorial, such as a large and growing supply of patients due to ageing, and insufficient capacity in hospitals due to personnel and resource shortages. Cardiovascular disease is the most common cause of mortality and morbidity, and as such contributes enormously to overcrowding. In 2019 there were an estimated 5.8 million new cases of ischemic heart disease in Europe [3]. Therefore, a large volume of patients presented to the hospital (around 1.95 million per year in the Netherlands [4]) are presented with symptoms of possible cardiac origin. However, not all patients visiting the ED need to be admitted to the hospital. For example, 80% of patients visiting the ED because of chest pain do not have an acute coronary syndrome (ACS) and can be reassured and discharged after a short analysis [5,6]. If these patients could be identified before visiting the ED, this could relieve pressure from EDs and prevent time-consuming and stressful ED visits for patients.

While there is extensive experience with prehospital triage in patients with trauma, the experience with prehospital triage in patients with cardiac symptoms is still limited. Recently, the FamouS Triage [7], ARTICA [8] and Hollands Midden Acute Regional Triage–cardiology [9,10] studies focused on improving triage of cardiac patients when patients contact the emergency medical services (EMS). These studies focused on selecting “low risk” patients who could safely stay at home after paramedic assessment. The FamouS and ARTICA studies used prehospital point-of-care troponin assessments, while the Hollands Midden Acute Regional Triage–cardiology study implemented a novel triage platform combining prehospital and hospital data.

Of note, the decision whether a patient can stay at home or should be transported to an ED in these studies was a purely human decision by health care professionals. The accuracy of these decisions is therefore highly dependent of training and expertise. Within these processes enormous amounts of data were gathered, processed, evaluated, and analyzed.

Artificial intelligence (AI) could be useful in analyzing data in medicine [11,12]. In cardiology, AI has mostly been used in integration and analysis of cardiovascular imaging [13]. However, there is potential to aid health care professionals in clinical decision-making such as certain apps do [14]. AI could be useful in making predictions or risk scores by learning from the available data. It might then be possible to identify low-risk patients through these AI generated risk scores in the prehospital setting. Patients could be reassured and safely stay at home, instead of being presented to the hospital.

The aim of this study was to develop an AI model able to predict ACS from prehospital data in patients presenting to the EMS. The AI model may be used as a proof of concept for future research on prehospital decision-making. In order to be a reliable tool, the AI model should have an increased specificity and at least a similar sensitivity as compared to regular care, as this could lead to an increase in patients staying at home after EMS consultation.

## Methods

### Study Design and Patient Population

The retrospective cohort study included all adults (aged 18 years or older) presenting to the regional EMS Hollands-Midden, servicing around 800,000 inhabitants in a mostly urban area, between September 2018 and September 2020 for symptoms suspected to be of cardiac origin. Patients were recruited in the prehospital setting by nurse paramedics. All data were acquired by a nurse paramedic and noted in AMBUFORMS (Topicus). Baseline characteristics are shown in Table 1.

Patients experiencing out-of-hospital cardiac arrest, (cardiac) shock, or patients visited by the EMS for noncardiac symptoms were excluded. The final diagnoses for ACS (defined as ST elevation myocardial infarction, non-ST elevation myocardial infarction, or unstable angina pectoris [15]) from all referrals to the ED were acquired through hospital billing data.

**Table 1:** Baseline characteristics of all recruited patients divided between patients who were ultimately presented to the hospital and patients who stayed at home after EMS^a^ consultation in this retrospective cohort study analyzing an AI algorithm in prehospital cardiac care.

Characteristic		Hospital (n=7386)	Home (n=72)
Women, n (%)		3991 (54)	37 (51)
Age (year), mean (SD)		68 (15)	67 (12)
Distance to hospital (km), mean (SD)		11.6 (8.3)	14.3 (12.0)
**Day of presentation, n (%)**			
	Monday	1414 (19)	20 (28)
	Tuesday	1412 (19)	13 (18)
	Wednesday	1224 (17)	15 (21)
	Thursday	1196 (16)	5 (7)
	Friday	1260 (17)	10 (14)
	Saturday	438 (6)	5 (7)
	Sunday	442 (6)	4 (6)
Chest pain at presentation, n (%)		2741 (37)	31 (43)
ACS^b^ diagnosis, n (%)		980 (13.3)	4 (5.6)

^a^EMS: emergency medical service.

^b^ACS: acute coronary syndrome.

### AI Model

Separate columns of data points, or features, were filled by paramedics for every patient. Patient data were stored on an external secure database AMBUFORMS. Patient data comprise quantitative data such as oxygen saturation, blood pressure, and heart rate and textual data created by the paramedic, such as the patient’s medical history, medication use, current symptoms, and physical examination. Table S1 in Multimedia Appendix 1 shows an overview of all available features evaluated by the AI model.

**Figure 1:**
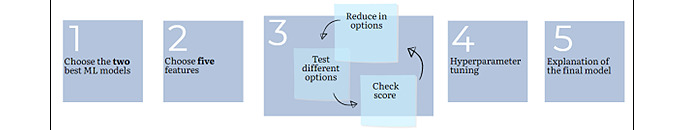
In total, there are 5 steps toward developing the final artificial intelligence (AI) model in this retrospective cohort study analyzing an AI algorithm in prehospital cardiac care. ML: machine learning.

The 5 steps toward developing the final AI model are shown in Figure 1. The model was developed using Python (version 3; Python Software Foundation). At first, a machine learning (ML) algorithm (or model) was chosen, afterwards the features needed were selected and then the model was tested and improved using iterative evaluation and in a further step through hyperparameter tuning. Finally, a method was selected to explain the final AI model.

In the first step, the *2* best ML models (or algorithms) were selected from 4 algorithms, namely support vector machine (SVM), random forest (RF), k-nearest neighbor (KNN) and logistic regression (LR). These models were preselected because they are well known when applying natural language processing (NLP) [16,17].

The *SVM model* converts the input to a vector in space. If all inputs are plotted, a hyperplane will be created. This plane is able to separate 2 classes of input from each other. The *RF model* is a classification algorithm consisting of many decisions trees. It creates an uncorrelated forest of decision trees from building individual decision trees. The forest of trees is more accurate than any individual tree. The *KNN model* finds distances between queries and examples in the data, selecting the specified number (K) closest to the query. Then the model votes for the most frequent label in the case of classification. The *LR algorithm* can be used for regression as well as classification tasks, for our model the classification tasks are used. LR has a binary response variable, which belongs to one of the classes. It is used to predict categorical variables with the help of dependent variables. Every model generated an Fβ score by analyzing all available data points. The most appropriate models were selected based on their respective Fβ score. The Fβ score was calculated as (1+β^2) × ((precision × recall) / ((β^2 × precision) + recall)).

Features (or columns of data) were selected in the second step. Further, 3 new features were created; a selection of all available data (CompiledALL), a selection of all textual data (CompiledTEXT), and a selection of all data thought to be relevant by a consulted cardiologist (CompiledSELECT). The CompiledSELECT feature was a combination of medical history, current symptoms, and electrocardiogram description, all of which were textual data noted by a nurse paramedic on the scene. Based on the Fβ and recall scores, commonly used within AI, the most relevant features were selected.

In the third step, separate parts of the algorithm were tested, after which highest scores were compared and other options were reduced or eliminated. In the first phase of this “loop” the 2 remaining models are tested, and the model with the lowest recall scores was eliminated. In the second phase, the selected features from step 2 were preprocessed and analyzed. The final feature was selected for the model. Then the threshold for the algorithm was analyzed and determined to find the correct false negative (FN) score.

The fourth step is fine-tuning the hyperparameters. In ML models settings can be altered to change the behavior of the model, and make predictions more accurate. Each model has one or multiple of these settings, called hyperparameters. For example, the SVM model has only 1 hyperparameter, named C. This hyperparameter has the options: 0.01, 0.1, 1, 10, 100, and 1000. During the previous steps, the default settings were used. By changing these settings, through trial-and-error, the final AI model can be optimized and the outcomes altered.

A *Python* package called “Explain Like I’m 5” (ELI5) was used to improve understanding of the model [18]. ELI5 explains classifiers and predictions in NLP by scoring the importance of words in text. The higher the importance of a word, the more influence it has on the eventual output of the AI model.

### Statistics

The following metrics were used to test reliability of the final AI model: precision, recall, and Fβ score. The Fβ score combines precision and recall, and the “beta” highlights the importance of one of the 2 metrics. A beta of 1 means both metrics were equally important, a beta lower than 1 means precision was more important, and a beta higher than 1 means recall was more important.

These metrics were clinically correlated using sensitivity, specificity, negative predictive value (NPV), and positive predictive value (PPV). Sensitivity (=recall) is the ability to correctly identify patients with a disease, in this case meaning no ACS were missed. Specificity is the ability to correctly identify patients without the disease, for the purpose of this study meaning no patients were unnecessarily presented to the hospital. NPV predicts the likelihood of a correct decision to leave patients at home and PPV (=precision) predicts the likelihood of a correct decision to present a patient to the hospital. The equations are given in Table S2 in Multimedia Appendix 2. All these parameters were calculated using true positives (*TP*), false positives (*FP*), true negatives (*TN*), and *FN*. *TP* was defined as a patient who was presented to the hospital who ultimately experienced ACS. *FP* was defined as a patient presented to the hospital who did not experience ACS. A *TN* was a patient who could stay at home and did not experience ACS, and a *FN* was defined as a patient who stayed at home but ultimately did experience ACS.

### Ethical Considerations

This study complies with the Declaration of Helsinki and the triage method was approved by the Hospital’s Medical Ethics Committee (P18.213). Patients were requested to provide verbal informed consent for participating in the triage method. Data were analyzed anonymously, all patient data were deidentified.

## Results

### Study Population

In total, 7458 patients (mean 68, SD 15 years, 54% men) were included in this study. For every patient, 270 features were available for the AI model. The primary presenting symptom was chest pain in 4686 (63%) patients, while 2772 (37%) patients had other symptoms (such as dyspnea, palpitations, or near-collapse). The EMS nurse paramedics decided that 72 patients could stay at home (1%): these patients were consequently not transported to the ED. Accordingly, in 7386 patients a medical analysis was performed at the ED: this showed an ACS in 980 patients. From the patients who stayed at home ultimately 4 were diagnosed with ACS within 30 days of staying at home.

### AI Model

The RF model had a mean Fβ score of 0.61, and the LR model had a mean Fβ score of 0.63. The 2 best models were the SVM model with an Fβ score of 0.71 and the KNN model with an Fβ of 0.88. The Fβ had a beta of 2 because this would mean that FN, an important outcome for the final model, had a higher weight. Table S3 in Multimedia Appendix 3 shows the outcomes from all AI models per feature.

Second, the 5 most relevant features were selected. The Fβ (again with a beta of 2) scores of all 17 features were between 0.75 and 0.89 for both the KNN and the SVM model. The recall scores were used to reduce the starting 17 features to 5. The “CompiledSELECT”-, physical examination–, physical survey–, differential diagnosis–, and control room note features had the highest recall scores. For the SVM model these were 0.6, 0.85, 0.71, 0.79, and 0.61, respectively. The KNN model had recall scores of 0.13, 0, 0.02, 0.01, and 0.05.

In the third step recall and FN score of both models for the 5 selected features were calculated. The recall scores for SVM were higher (as seen in step 2) and therefore the KNN model was eliminated. In the second phase the 5 remaining features were preprocessed, reducing the model to 1 single feature. The feature with the highest recall and FN score was “CompiledSELECT.” Lastly a threshold was selected for the model. A threshold of 0.955, 0.983, and 0.991 gave recall scores of 0.95, 0.995, and 1, respectively.

The final step of the model, step 4, is the tuning of hyperparameters. As mentioned before, the SVM model has 1 hyperparameter, or setting, named C. Recall scores were highest when this hyperparameter was set to 0.1.

**Figure 2:**
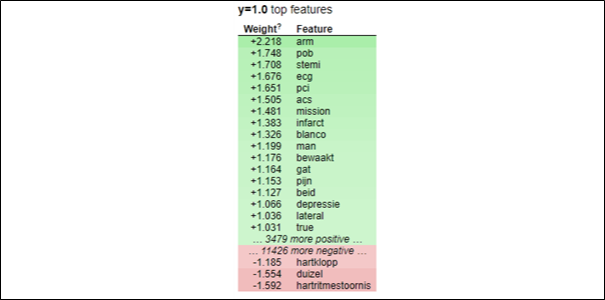
Global explanation of the artificial intelligence (AI) model in this retrospective cohort study analyzing an AI algorithm in prehospital cardiac care. Green shows words which correlate with patients who experienced myocardial infarction, whereas red correlates with patients who did not experience myocardial infarction. acs: acute coronary syndrome; ecg: electrocardiogram; pci: percutaneous coronary intervention; pob: chest pain; STEMI: ST-elevation myocardial infarction.

**Figure 3:**
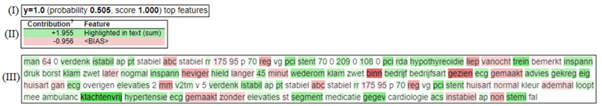
Explanation by ELI5 where red words represent the class “myocardial infarction” and green “no-myocardial infarction” in this retrospective cohort study analyzing an AI algorithm in prehospital cardiac care. ELI5: “Explain Like I’m 5.”.

The results from analyzing the textual data by the final AI model were shown through ELI5 in Figures 2 and 3. The AI model recognized words that are linked to myocardial infarction in green and words that are not linked to myocardial infarction in red. All textual data were analyzed this way. The final AI model resulted in a recall score of 1.00 and precision score of 0.15 as compared to 1.00 and 0.12 in usual care respectively.

### Clinical Results

The AI model was able to identify 713 TNs (patients who could stay at home without experiencing ACS) as compared to 68 TNs in usual care. There were 4 FNs (patients who stayed at home but did experience ACS) in the usual care group and 4 in the AI model, meaning a total of 645 patients could potentially stay at home without missing more ACS. This is an increase in TNs of 945% (n=577). Subsequently, the FPs (patients presented at the hospital without experiencing ACS) decreased by 10% (n=645) as there were 5761 patients identified in the AI model and 6406 patients in usual care. TPs remained similar when comparing usual care with the AI model, both comprised of 980 patients.

The AI model had a specificity of 11% and a sensitivity of 99.5% whereas usual care had a specificity of 1% and a sensitivity of 99.5%. The PPV of the AI model was 15% and the NPV was 99%. The PPV of usual care was 13% and the NPV was 94%.

## Discussion

### Principal Findings

This study evaluates a newly developed AI model to predict ACS in patients presenting to the EMS. The model was developed as a proof of concept for prehospital triage based on large amounts of EMS data. This study demonstrates that the AI model is able to predict ACS with a similar sensitivity and a higher specificity as compared to usual care, which means more patients can stay at home and a low number of ACS are missed.

Resources in health care are scarce, the shortages in health care personnel are increasing and hospitals and ED’s are increasingly (at risk of being) overcrowded. With an ageing population more patients are expected in the near future, putting even more strain on the existing health care resources. It is of utmost importance to correctly allocate (or triage) these scarce resources to the right patient, preferably as early as possible in the health care process, thus, ideally, before patients are presented to the ED. Selecting the appropriate patient to safely stay at home can prevent stressful and time-consuming ED visits for patients. Risk scores developed and analyzed by AI could be useful since the current forms of prehospital triage [8,9,19-21] all depend on health care personnel, such as EMS paramedics, cardiologists, or general practitioners. AI models could reduce costs and decrease the amount of personnel needed while maintaining high quality health care. As a last point, (human) experience-based triage has the potential for errors, whereas AI, by definition, has a lower interobserver variability. Implementation of AI may therefore potentially limit these errors.

AI within the field of cardiology has mainly been applied in automating the interpretation of cardiac imaging [13,22] and electrocardiography [23]. It has also proven to be useful in predicting events in asymptomatic patients and in patients following ACS [24,25], or, when using textual data, by determining cardiovascular disease risk from social media [26]. In-hospital AI, outside of the field of cardiology, has been able to identify patients admitted to the ED at risk of clinical deterioration [27], and identify low-severity patients for quick discharge [28]. However, the evidence for the use in prehospital triage is scarce. In the prehospital setting, there have been some studies where AI was able to predict the need for critical care or hospital admission for all patients [29,30], and in mass casualty incidents [31]. However, prehospital triage for cardiac symptoms with the intention for patients to stay at home after EMS consultation has not been described.

Because of the retrospective nature of this study, the binary outcome (ACS or no ACS) was known and thus supervised ML classification was used. The 4 presented algorithms (SVM, RF, KNN, and LR) are most commonly used [16,17] when using supervised classification and analyzing textual data (and thus when applying NLP). Ultimately, the SVM algorithm was implemented in the final AI model.

The model had a specificity of 11% and a sensitivity of 99.5% whereas usual care had a specificity of only 1% and a sensitivity of 99.5%. The AI model led to an 1100% increase in specificity as compared to regular care. The AI model was able to identify more TPs, meaning more patients without ACS could stay at home after EMS consultation. Both methods had a very high sensitivity, meaning there were (almost) no FNs. Thus, a very small number of patients (<0.5% or n=4) were left at home who ultimately experienced ACS. This is important, as delayed care in patients with ACS results in higher mortality and disability rates [32]. To the best of our knowledge there are no examples of studies within the field of cardiology that describe the specificity and sensitivity of the clinical use of their AI algorithm as described in this study. As mentioned above, in clinical practice the specificity of prehospital triage for cardiac symptoms is very low, as ACS is a diagnosis that is not to be missed. Of note, sensitivity and specificity of all patients in this study, low-risk, medium-risk and high-risk combined, are comparable to the sensitivity and specificity of only the low-risk patients in the HEART score (<2), which had a sensitivity of 98.9% and a specificity of 14.7% [33].

This study is the first study to evaluate an AI model in prehospital triage of cardiac patients. The model analyzed routinely collected data from prehospital EMS care and, when applied, could be a useful tool to aid in triage for first responders. The AI model could easily be trained for other purposes, such as different symptoms or cardiac symptoms in different countries. An AI based model is futureproof, since, when available, more advanced techniques, models, and approaches could be built in to the model. The complexity and amount of medical data (and patients) is expected to increase in the future; therefore, advanced pattern finding by AI can be hugely beneficial. It seems that an AI model which uses text classification could be useful for other medical specialties as well. Prehospital triage of surgical patients could possibly be improved by an AI model. The model could analyze the textual data from a nurse paramedic and assess whether patients need to be transported and, importantly, which hospital might be best suited for that specific patient. For instance, patients with fever and specific abdominal pain could be presented to a hospital with surgical capabilities, where patients with shortness of breath and a history of coughing up blood could be assessed in a hospital with the capabilities of treatment of pulmonary embolism, thereby improving prehospital triage. Furthermore, it is far less dependent on the (scarcely) available professional workforce. For future research, validation, and eventual implementation it is important to streamline the methods of data collection and analysis. By structuring medical data AI models could be of even greater benefit. Of importance, health care professionals should always have the final say in the decision.

This study has some limitations. The most important limitation is that the AI model was only able to predict ACS or “no ACS,” a sort of pseudo-diagnosis. It does not take into account important, possible life-threatening causes of chest pain, such as pulmonary embolism or aortic dissection. Therefore, it cannot be said with surety that patients can be left at home if ACS is ruled out. Future research should include all of these possible causes and look for stronger end points such as hospital admission for other causes or even 30-day mortality. It is important to note that an AI model should always be used as a tool, or aid, in prehospital decision-making. It should never be used to overrule decisions made by clinicians who are with the patients.

Furthermore, the AI model has only been able to identify patients in a retrospective manner, validation and further research is needed in a prospective setting. Herein also lies the practical limitation, as it is still very difficult to prospectively validate AI models, especially in the prehospital setting. Furthermore, the model needs to be trained regularly and there will always be cases which the model hasn’t seen before making it possibly prone to errors.

### Conclusions

This retrospective study is a proof-of-concept of an AI model which was developed to identify patients with ACS in the prehospital setting based on textual data. The model had a similar sensitivity and an 1100% increased specificity as compared to usual care.

## Appendix

Multimedia Appendix 1 Table S1. List of features used in the AI model, originally in Dutch translated to English.

Multimedia Appendix 2 Table S2. Equations of metrics used in the presented study.

Multimedia Appendix 3 Table S3. Fβ score of the four different models per Dutch feature (translation in English) used in the AI algorithm.

## Abbreviations

ACS: acute coronary syndrome
AI: artificial intelligence
ED: emergency department
ELI5: Explain Like I’m 5
EMS: emergency medical services
FN: false negative
FP: false positive
KNN: k-nearest neighbor
LR: Logistic Regression
ML: machine learning
NLP: natural language processing
NPV: negative predictive value
PPV: positive predictive value
RF: Random Forest
SVM: support vector machine
TN: true negative
TP: true positive

## Data Availability

The data sets generated or analyzed during this study are available from the corresponding author on reasonable request.
