# Supplementary material for: AI Algorithm to Predict Acute Coronary Syndrome in Prehospital Cardiac Care: Retrospective Cohort Study
Source: JMIR Cardio. 2023 Oct 31;7:e51375. doi: 10.2196/51375 (PMC10646678; doi:10.2196/51375)
Supplement: Multimedia Appendix 1 [file cardio_v7i1e51375_app1.docx]

**
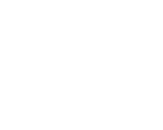
Table S1.** List of features used in the AI model, originally in Dutch translated to English.

Trip number

Trip date

Month

Year

Day of the week

Triage hospital

Name

Gender

Age

Date of birth

Telephone

BSN

GP

RetrievalAddress

RetrievelAddressNr

Zipcode

Location

DeliveryAddress

DeliveryAddressNr

Zipcode

Location.1

DeliveryDescription

Distance

EMS Control Room Notes

EMS Control Room Severity score

Custom urgency

Report time

Turn-off time

Departure Time

ArrivalIncident

DepartureToHospital

ArrivalAtHospital

AmbuAvailable

EndTrip

Out of office hours

Chestpain

Palpitations

Dyspnea

Collaps

ICD/Pacemaker

Resuscitation

OVERVIEW

OTHER

CARDIAC PATIENT

Weekend

Evening

Planned transfer

Remark on transfer

Type of transport

Caller on site

Discussed with GP

Type of accident

Airway

Breathing

Saturation

Respiration

Oxygenating mask

Oxygenating mask l/min

Oxygen non-rebreathing mask

Oxygenizing CPAP mask

Circulation

Pulse

Systolic BP

Diastolic BP

Capillary refill extended

Pale

Cold, clammy, sweating

Type of rhythm

Explanation ECG

Cardioversion

Defibrillation

Circulation other procedures

ECG

Disability

Duration or loss of consciousness

Pupils


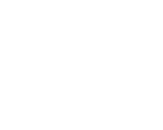
Left pupil

Right pupil

Glucose

Temperature

Exposure

Medication

Medication description

Past

Past description

Event prior notice

Prior Announcement

Event description

Reason for reporting

Inspection

Medical history

Medical history per organ

Physical examination

Patient Deceased

Working diagnosis arrhythmia

Working diagnosis ACS

Working diagnosis resuscitation

Working diagnosis medical specialty

Working diagnosis injury code

Diagnosis cardiology

Secondary diagnosis: neurology

Internal medicine

Secondary diagnosis intoxication

Secondary diagnosis paediatrics

Secondary diagnosis psychiatry

Secondary diagnosis pulmonology

Secondary diagnosis traumatology or surgery

Specialist diagnosis obstetrics

Secondary diagnosis: revalidation

Explanation of treatment/follow-up care

Resuscitation

Differential diagnosis considerations

Treatment - Intubation

Treatment - Ventilation

Treatment - Infusion

Treatment - Medication

Treatment - Resuscitation

Clinical picture - Nausea

Clinical picture - Shock/bleeding

Clinical picture - Chest pain

Clinical picture - Collapse

Clinical picture - Acute abdomen

Clinical picture - Loss of consciousness

Clinical picture - Insult

Clinical picture - Headache

Clinical picture - Fever

Clinical picture - Intoxication

Hypo-hyperthermia

Pain in the back or flank

Obstetric Syndrome

Clinical picture - Psychological disorder

Non-Trauma Treatment - Intubation

Non-Trauma Treatment - Ventilation

Non Trauma Treatment - Infusion

Non Trauma Treatment - Medication

Non Trauma Treatment - Resuscitation

Destination

Reception type

Reported arrival time

Arrival time

Details

ACS
