# Supplementary material for: AI Algorithm to Predict Acute Coronary Syndrome in Prehospital Cardiac Care: Retrospective Cohort Study
Source: JMIR Cardio. 2023 Oct 31;7:e51375. doi: 10.2196/51375 (PMC10646678; doi:10.2196/51375)
Supplement: Multimedia Appendix 2 [file cardio_v7i1e51375_app2.docx]

Table S2. Equations of metrics used in the presented study.

| Sensitivity (or *recall*) | $\frac{tp}{tp+fn}$ |
| --- | --- |
| Specificity | $\frac{tn}{tn+fp}$ |
| NPV | $\frac{tn}{tn+fn}$ |
| PPV  (or precision) | $\frac{tp}{tp+fp}$ |
| Fβ score | $\left( 1+\beta^{2} \right)\times\frac{precision \times recall}{\left( \beta^{2}\times precision \right)+recall}$  or  $\frac{\left( 1+\beta^{2} \right)\times tp}{\left( 1+\beta^{2} \right)\times tp+\beta^{2}\times fn+fp}$ |

*Footnote: tp: true positives, fp: false positives, tn: true negatives, fn: false negatives*
