# Supplementary material for: AI Algorithm to Predict Acute Coronary Syndrome in Prehospital Cardiac Care: Retrospective Cohort Study
Source: JMIR Cardio. 2023 Oct 31;7:e51375. doi: 10.2196/51375 (PMC10646678; doi:10.2196/51375)
Supplement: Multimedia Appendix 3 [file cardio_v7i1e51375_app3.docx]

**Table S3.** Fβ score of the four different models per Dutch feature (translation in English) used in the AI algorithm.

| Features: | SVM | RF | KNN | LR |
| --- | --- | --- | --- | --- |
| MedischKladblokMKA (EMS Control Room Notes) | 0.75067 | 0.656166 | 0.865952 | 0.754692 |
| Toelichting ECG (Explanation ECG) | 0.80496 | 0.779491 | 0.884718 | 0.787534 |
| Exposure | 0.864611 | 0.186327 | 0.882708 | 0.193029 |
| Medicatie omschrijving (Medication description) | 0.543566 | 0.468499 | 0.878686 | 0.544906 |
| Past omschrijving (Past description) | 0.704424 | 0.466488 | 0.881367 | 0.710456 |
| Event omschrijving (Event description) | 0.792895 | 0.733914 | 0.882038 | 0.786863 |
| Reden van melding (Reason for reporting) | 0.701743 | 0.717828 | 0.869303 | 0.709786 |
| Schouw (Inspection) | 0.438338 | 0.428284 | 0.878016 | 0.455094 |
| Anamnese (Medical history) | 0.847855 | 0.823056 | 0.882708 | 0.835121 |
| Tractus anamneses (Medical history per organ) | 0.871984 | 0.185657 | 0.882708 | 0.199732 |
| Lichamelijk onderzoek (Physical examination) | 0.262735 | 0.838472 | 0.880697 | 0.260054 |
| Toelichting behandeling (Explanation treatment) | 0.870643 | 0.840483 | 0.88941 | 0.865952 |
| Overwegingen (Considerations) | 0.290214 | 0.270107 | 0.882708 | 0.301609 |
| Bijzonderheden (Details) | 0.867292 | 0.865952 | 0.883378 | 0.865952 |
| CompiledALL | 0.817694 | 0.715818 | 0.869303 | 0.8063 |
| CompiledTEXT | 0.813673 | 0.727212 | 0.867292 | 0.813673 |
| CompiledSELECT | 0.816354 | 0.66555 | 0.868633 | 0.80563 |
| Mean Fβ score: | 0.709391 | 0.609959 | 0.878213 | 0.629199 |
